# Phoenix from the ashes: dramatic improvement in severe late-onset methylenetetrahydrofolate reductase (MTHFR) deficiency with a complete loss of vision

**DOI:** 10.1007/s00415-021-10841-x

**Published:** 2021-10-17

**Authors:** Anne-Sophie Biesalski, Sabine Hoffjan, Ruth Schneider, Huu Phuc Nguyen, Gabriele Dekomien, Thomas Lücke, Christiane Schneider-Gold, Britta Matusche, Ralf Gold, Ilya Ayzenberg

**Affiliations:** 1 Department of Neurology, St. Josef Hospital, Gudrunstraße 56, 44791 Bochum, Germany; 2 Department of Human Genetics, Ruhr-University, Bochum, Germany; 3 University Children’s Hospital, St. Josef Hospital, Bochum, Germany; 4 Center for Rare Diseases Ruhr (CeSER), Bochum, Germany; 5 Department of Neurology, Sechenov First Moscow State Medical University, Moscow, Russia; 6 Institute of Neuroradiology, St. Josef Hospital, Ruhr University Bochum, Bochum, Germany

Dear Sirs,

Severe methyltetrahydrofolate (MTHFR) deficiency is a rare autosomal recessive demyelinating disease with a wide range of neurological symptoms [1]. MTHFR plays an important role as a methyl donor for the methylation of homocysteine to methionine. MTHFR catalyses the reduction of 5,10-methylenetetrahydrofolate to 5-methyltetrahydrofolate which serves as a methyl donor in the remethylation of homocysteine to methionine and is the most common form of folate in blood, cerebrospinal fluid and tissues. MTHFR deficiency leads to a partial or complete lack of methylation of homocysteine to methionine resulting in highly elevated plasma total homocysteine and low plasma methionine. Especially low methionine level causes a depletion of *S*-adenosylmethionine which plays an important role as a donor in methylation reactions in the central nervous system. In cases of inborn defects of 5,10-MTHFR the residual enzyme activity corresponds to the severity of symptoms as well as to the age at disease onset [1–4]. Early treatment with betaine, a methyl donor, has been shown to prevent mortality and improves psychomotor development even in pediatric cases with severe enzyme deficiency [5–7].

Severe MTHFR deficiency usually occurs shortly after birth or in early childhood with failure to thrive, muscle hypotonia and hydrocephalus or apnea. Late onset is characterized by predominantly psychiatric or cognitive symptoms, seizures, and—in nearly all cases—gait disorders [3, 8–10].

We report a 30-year-old female patient of Lebanese origin from a consanguineous family. At the age of 29 she suffered from a subacute decrease in visual acuity, leading to complete loss of vision (no light perception) within 8 weeks. During the following 6 months she developed severe spastic tetraparesis and cognitive impairment with pronounced bradyphrenia and therapy refractory seizures. At the timepoint when the patient first came to our clinic, she remained blind, bedridden and permanently dependent on support in all her daily activities for about 16 months. The clinical examination showed in particular a normal direct and consensual pupillary reflex.

MR-imaging revealed symmetrical paraventricular leukoencephalopathy as well as moderate brain atrophy (Fig. 1). The ophthalmological examinations initially confirmed a complete visual loss.

**Fig. 1:**
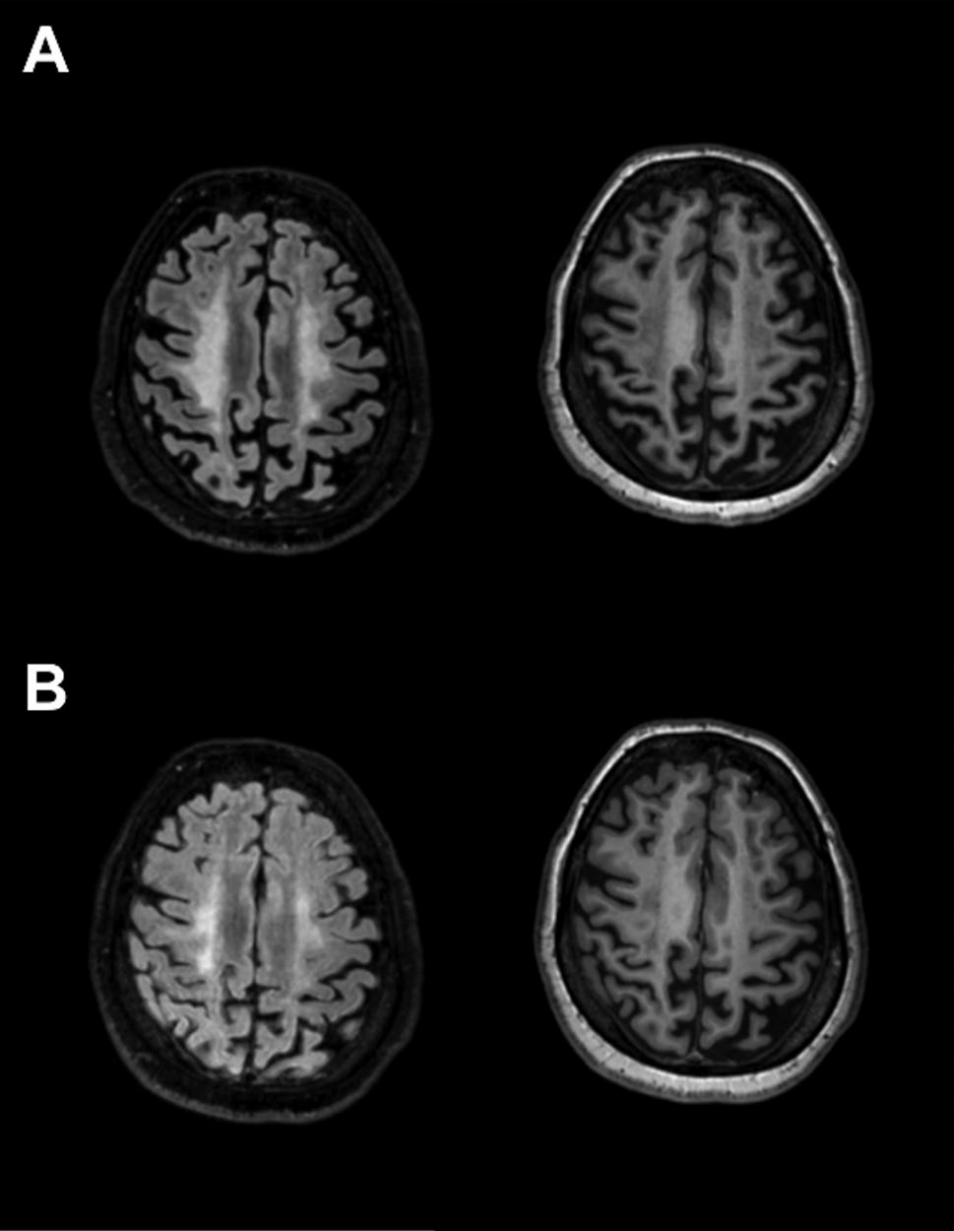
Baseline and follow-up MRI after 3 month of the index patient. Axial T2-weighted FLAIR (left) and corresponding T1-weighted (right) image of the index-patient at baseline (**A**) and follow-up (**B**) investigation showing substantial regression of the hyperintense (T2), respectively, hypointense (T1) areas, reflecting lesion volume reduction, which could be objectified by LPA-based lesion quantification (baseline 62.11 ml and follow-up 21.69 ml T2 lesion volume)

The genetic panel test identified a homozygous mutation c.973C > T,p (Arg325Cys) in the MTHFR gene. Accordingly, the blood test showed hyperhomocysteinemia (256.1 μmol/l; reference < 9 μmol/l) at normal methionine levels, confirming MTHFR deficiency.

We started a high-dose oral therapy with betaine (betainhydrochloride 10 g/day), methionine, vitamin B12, B6 and folic acid. Significant improvements in motor and cognitive functions were observed within 4–6 days of therapy. The patient also reported improved visual perception within only 2 weeks. First available VEP demonstrated severely prolonged P100-Latency (Table 1). Surprisingly, the optical coherence tomography (OCT) did not show any pathologies (Table 1). At 6-week-follow-up, she showed an almost complete recovery of vision (Table 1) and almost complete regeneration in cognitive abilities. Her gait improved significantly, enabling her to walk 20 m without help or assistance. Homocysteine level decreased to 66.0 μmol/l. Three months follow-up MRI surprisingly showed a notable reduction of paraventricular leukoencephalopathy with regressive hyperintense T2 lesions (Fig. 1).

**Table 1:**
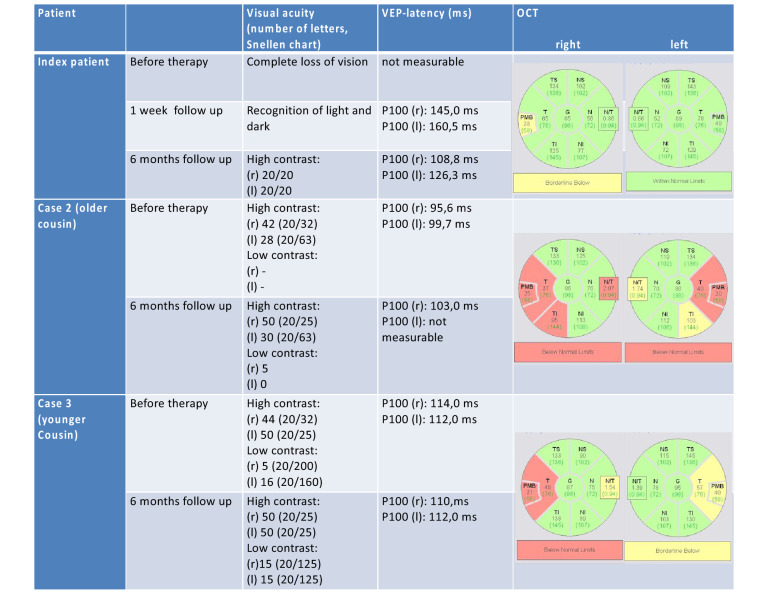
Comparison of visual acuity and retinal degeneration in OCT of the index patient and two further family members with delayed treatment start

Index patient demonstrated a complete restoration of her visual acuity in parallel to normalization of P100 latency without any relevant neuroaxonal retinal degeneration. In contrast, we observed only a mild functional improvement with profound temporal RNFL atrophy and normal P100 latencies in the two cousins, confirming substantial neuroaxonal damage.

*RNFL* retinal nerve fiber layer, *OCT* optical coherence tomography

In the course of time we also treated two cousins of our index patient, who suffered from the same genetic mutation. The older cousin (35 years old, Table 1) was dependent on a wheelchair for already 6 years. The neurological examination showed a spastic paraparesis and mild cognitive impairment. Ophthalmological examination revealed moderately reduced visual acuity with bilateral temporal atrophy of the retinal nerve fiber layer (RNFL) in the OCT (Table 1). After 6 months of high-dose betaine therapy, visual acuity improved in the right eye, although there were no changes in the VEP (Table 1). The pre-existing paraparesis and cognitive impairment remained unchanged.

The younger cousin suffered from an advanced paraparesis for 7 years and was dependent on crutches. Visual acuity was significantly reduced, OCT showed mild temporal atrophy on the right (Table 1), VEP showed normal P100 latency by substantially decreased amplitudes. After 6 months of betaine therapy, this patient showed minimal improvement of the visual acuity without changes in VEP and OCT, as well.

Unique to this case series is the complete—and completely reversible—loss of vision in the index patient, as well as some improvement of the visual acuity in two other family members many years after disease manifestation. Visual loss seems to be a rare initial manifestation in late-onset MTHFR deficiency and has only been reported in isolated cases [3, 11]. One probable cause of the subacute progressive loss of vision in this case could be disrupted optic nerve myelination due to central deficiency in *S*-adenosylmethionine [12]. Severely prolonged P100-latencies confirm this hypothesis, however, pupillary light reflex was normal and no neuroaxonal retinal atrophy developed later. Alternatively, reversible central blindness could be a plausible explanation of the complete recovery of the visual function in parallel to the improvement of other cognitive functions. Posterior reversible encephalopathy syndrome (PRES) could not be confirmed either in the MRI or based on the course of the disease (slowly progressive deterioration of visual acuity and a good response to betaine therapy). In contrast, only minimal improvement could be achieved in both cousins due to a long disease course and irreversible neuroaxonal degeneration. The early high-dose betaine therapy in our index patient resulted also in impressive regression of the T2 lesion volume simultaneously with improvement of the latency of visual evoked potentials, indicating a remyelination revealed by both modalities (Table 1).

In conclusion, our case series confirms late-onset MTHFR deficiency as an important treatable genetic disease. Neurologists as well as ophthalmologists should be aware of this rare differential diagnosis in progressive visual loss without evidence of an ocular cause. Homocysteine screening should be considered as a relevant diagnostic test in unclear demyelinating leukoencephalopathy and subacute or progressive visual loss. Good visual recovery can be achieved if treated early before secondary axonal degeneration occurs.

## Data Availability

The datasets generated during and/or analysed during the current study are available from the corresponding author on reasonable request.
